# Application of Transformers to Chemical Synthesis

**DOI:** 10.3390/molecules30030493

**Published:** 2025-01-23

**Authors:** Dong Jin, Yuli Liang, Zihao Xiong, Xiaojie Yang, Haifeng Wang, Jie Zeng, Shuangxi Gu

**Affiliations:** 1 School of Chemical Engineering & Pharmacy, Pharmaceutical Research Institute, Wuhan Institute of Technology, Wuhan 430205, China; jindong0833@163.com (D.J.); 13104930276@163.com (Y.L.); 15107360517@163.com (Z.X.); skytacle@139.com (H.W.); 2 Hubei Key Laboratory of Radiation Chemistry and Functional Materials, School of Nuclear Technology and Chemistry & Biology, Hubei University of Science and Technology, Xianning 437100, China; mailyangxiaojie@126.com

**Keywords:** Transformer, reaction prediction, retrosynthesis prediction, SMILES, molecular graph

## Abstract

Efficient chemical synthesis is critical for the production of organic chemicals, particularly in the pharmaceutical industry. Leveraging machine learning to predict chemical synthesis and improve the development efficiency has become a significant research focus in modern chemistry. Among various machine learning models, the Transformer, a leading model in natural language processing, has revolutionized numerous fields due to its powerful feature-extraction and representation-learning capabilities. Recent applications demonstrated that Transformer models can also significantly enhance the performance in chemical synthesis tasks, particularly in reaction prediction and retrosynthetic planning. This article provides a comprehensive review of the applications and innovations of Transformer models in the qualitative prediction tasks of chemical synthesis, with a focus on technical approaches, performance advantages, and the challenges associated with applying the Transformer architecture to chemical reactions. Furthermore, we discuss the future directions for improving the applications of Transformer models in chemical synthesis.

## 1. Introduction

Chemical synthesis, as a core aspect of drug and material research and development, is directly related to the design, construction, and eventual application value of drug and material molecules [1,2]. However, the traditional, high-cost, trial-and-error approach to chemical synthesis often faces numerous challenges, including the multistep complexity of reaction processes, high selectivity and efficiency requirements, and the need for sustainable development aligned with the principles of green chemistry [3]. The development of computer-aided synthetic planning has led to significant advances in chemical synthesis technology [4]. In recent years, the application of machine learning-based methods, particularly deep learning, in chemical synthesis has expanded dramatically. With its powerful data-processing and pattern-recognition capabilities, it has provided new technological tools for molecular design [5,6], reaction prediction [7,8], and pathway planning [9,10]. These advancements have enhanced the accuracy of chemical reaction predictions, improved the efficiency of synthetic pathway planning, and fostered the development of intelligent automation, thereby providing crucial support for green chemistry and high-throughput experiments. Through diverse linear or graphical representations of molecular structures, some advanced learning architectures can capture molecular information, such as recurrent neural networks (RNNs), convolutional neural networks (CNNs), or graph neural networks (GNNs), are gradually being introduced to the task of modeling and learning from chemical data [11,12,13,14]. In the field of chemical synthesis prediction, researchers are primarily focused on understanding the formation of products and by-products. From the qualitative perspective, a critical aspect of chemical synthesis prediction is analyzing the structural transformations of products before and after the reaction. However, this process often relies heavily on expert empirical knowledge, making the development of effective machine learning methods for chemical data a significant challenge.

Along with the growing research on large language models (LLMs), these natural language processing (NLP) models were also widely applied in the field of chemical synthesis research [15,16]. Inspired by the success of NLP models in translation and generation tasks, researchers framed chemical reaction prediction and retrosynthetic pathway planning as tasks involving sentences composed of molecular “words”, treating them as linguistic string sequencing problems. The Transformer architecture, one of the most prominent LLMs, with its powerful sequence-modeling capabilities and parallel-computing advantages, has emerged as a key tool for these tasks [17]. In comparison with traditional RNNs used for natural language modeling, the Transformer model addresses issues like gradient vanishing and computational inefficiency that often arise when an RNN handles long sequential tasks. This is due to its unique self-attention mechanism, which enables it to capture global dependencies across long sequences [18]. This suggests that it is uniquely adapted to the current treatment of such qualitative synthetic prediction and long-range problems, such as chemical reaction prediction and inverse synthetic pathway planning [19].

**Figure 1:**
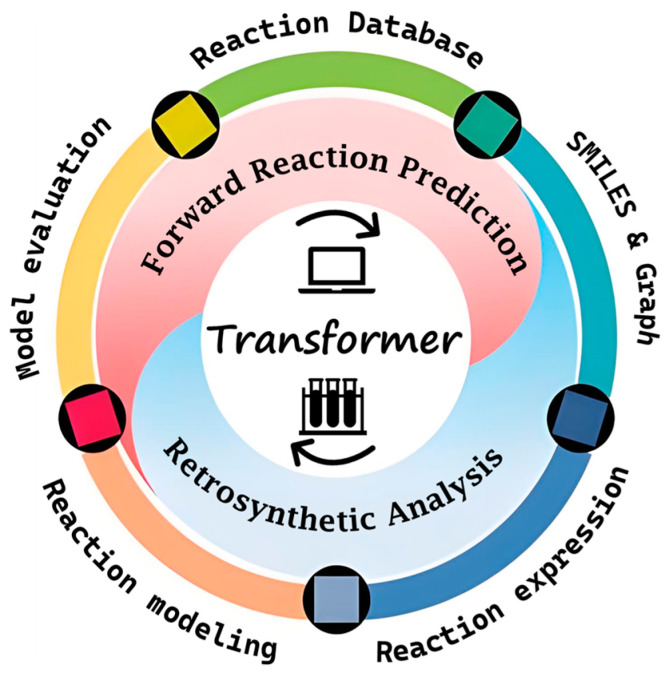
Schematic of the reaction and retrosynthesis applications of the Transformers.

In recent years, research on the chemical synthesis of Transformer-based structures has increased significantly. To effectively evaluate the progress in this field, we conducted a comprehensive review of the key previous studies. This review aimed to summarize the main applications and advances of Transformer models in chemical reactions; classify the datasets, model architectures, and technical approaches employed in qualitative reaction prediction studies; evaluate their performances in various tasks; and explore the challenges and future directions for these models in chemistry (Figure 1).

## 2. Task Adaptation and Reaction Dataset

### 2.1. Adaptability of Transformer

Chemical molecules are often represented using SMILES (Simplified Molecular Input Line Entry System), a notation system for linearizing molecular structures [20]. SMILES can also be used to represent chemical reaction equations (e.g., “reactants → agents → products”). Therefore, chemistry can be represented as a string of SMILES, forming words and sentences. At the core of the Transformer model is the self-attention mechanism, which dynamically assigns weights to each element in an input sequence. This mechanism captures global dependencies between any two positions in the sequence, allowing for better modeling of the contextual information, especially for long-sequence tasks [17]. The advantages of this mechanism in chemical synthesis are primarily the following: (1) Molecular dependency modeling: In chemical molecules, interactions between atoms (e.g., bonding relationships or electronic effects) may span long distances. Self-attention mechanisms can directly capture these long-range dependencies, enabling the accurate mapping of reactants to products. (2) Global influence of reaction conditions: The outcome of a chemical reaction is often significantly influenced by the reaction conditions (e.g., catalyst, temperature, pressure). By incorporating this condition information, the self-attention mechanism can capture the global effects of reaction conditions on molecular reaction pathways [21]. It is worth noting that in catalytic reactions, data-driven deep learning algorithms usually input catalysts as feature values of the reaction, along with the reaction conditions, or model them as molecules for reaction embedding, whereas common standard catalysts (e.g., Pd, Ni, Cu), whose mechanism of action and effects are usually fixed and well defined in some obvious standard catalytic reactions, may be used as a label for distinguishing reaction types [22,23].

In chemical reaction prediction, the Transformer’s self-attention mechanism helps analyze synergistic relationships between reactants to accurately predict complex reaction products [17]. In retrosynthesis planning tasks, a Transformer can make a prediction based on templates or not, and in the pursuit of predicting unknown reactions, it is more likely to be used for template-free prediction [24,25,26]. In addition, the Transformer combines search algorithms with retrosynthesis planning to generate efficient synthetic pathways, making it an important tool for chemical reaction prediction [27]. Meanwhile, the Transformer model is not limited to the SMILES representation. It can be combined with molecular maps to further capture the internal topology of molecules, making full use of the synergy between a GNN and a Transformer, a combination that greatly improves the modeling accuracy [28].

### 2.2. Architecture of Transformer

**Figure 2:**
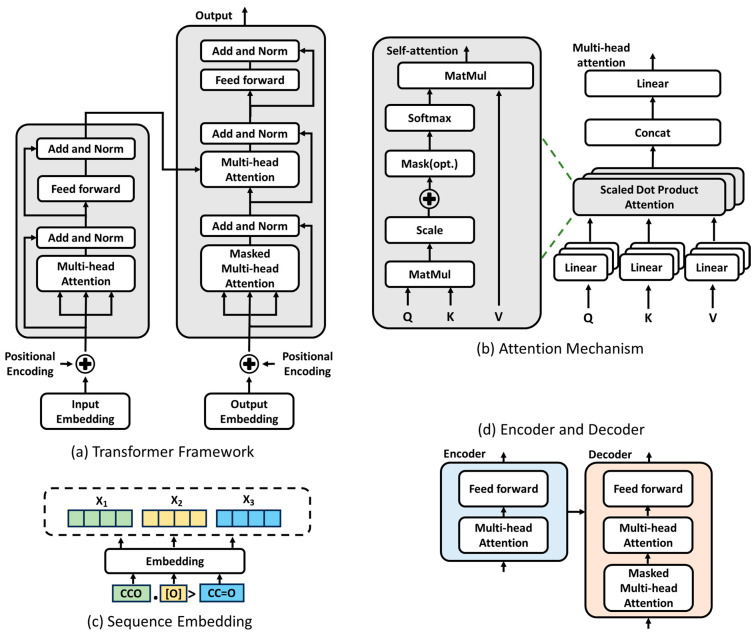
Architectural components of the Transformer model.

The Transformer is a deep learning model architecture designed for processing sequence data, first introduced by Vaswani et al. in 2017 [18]. The Transformer architecture comprises two primary components: the encoder and the decoder. Each component is built from a stack of identical modules, as illustrated in Figure 2a.

The encoder is composed of multiple stacked encoder layers, as shown in Figure 2d. Each encoder layer consists of three sub-modules: (1) multi-head self-attention, which captures relationships between each position in the input sequence and other positions; (2) a feed-forward neural network (FFN) that applies a nonlinear transformation to the output of the attention mechanism, enhancing the model’s expressive power; and (3) residual connection and layer normalization (RCLN), which improves the network trainability and gradient stability through skip connections and normalization [29]. The input into each encoding layer is a vector that embeds the input sequence into a fixed dimension using positional encoding to retain sequence order information. The output is a hidden representation of the sequence that captures contextual information for each position.

The decoder consists of multiple stacked decoder layers, as shown in Figure 2d, and is mostly different from the encoder in that the decoder incorporates masked multi-head self-attention, which allows the decoder to focus on the past position of the generated output to ensure the causality of the generated sequence, and encoder–decoder attention, which interacts the current state of the decoder with the encoder’s output and extracts global information of the input sequence from the encoder. The input into the decoder is the generated portion of the target sequence, along with the positional encoding, and the output is the next sequence in the stepwise generation of the target sequence, where the probability distribution of each possible output is computed by means of the softmax function. Compared with traditional RNN and long short-term memory (LSTM) [30] models, the Transformer does not rely on the sequential processing of sequences, but processes the entire sequence at the same time, which makes a big step forward in sequence modeling, and its encoder captures the global contextual relationships in the input sequence through the self-attention mechanism, while the decoder extracts the key information of the input sequence from the encoder, which is very suitable for processing complex long-sequence tasks, such as machine translation, text summarization, and chemical reaction prediction [31].

The self-attention mechanism is the core technology of a Transformer, as shown in Figure 2b, which allows the model to capture the global dependencies between positions in a sequence while processing the sequence. The self-attention mechanism is a weight assignment mechanism that dynamically adjusts the contribution of each position to the current task by calculating the correlation between each position (e.g., a word or an atom) in the sequence and all the other positions. The input into the self-attention mechanism is a matrix of three vectors: the query vector *Q* (the attention of each position to the other positions), the key vector *K* (the features of the other positions), and the value vector *V* (the actual information to be output). The similarity between the vectors *Q* and *K* is calculated by a dot product, which measures the relevance between the query and the key to obtain the attention score, with the following relation formula:

(1) $$Attention\left(Q,K,V\right)=Softmax\left(\frac{{QK}^{T}}{\sqrt{d_{k}}}\right)V$$

where $d_{k}$ is the dimension of the key vector, which is used to scale the size of the dot product to avoid unstable values. The resulting attention scores are then normalized by the softmax function to generate a weight distribution, which is then weighted and summed over the value vector *V* using the weights to generate the output representation. Each position in the encoder captures the global semantics of the sequence by paying attention to other positions in the sequence through the self-attention mechanism, while in the decoding process, the self-attention mechanism ensures that the current generated position can only pay attention to the past generated sequences to maintain causality, and the decoder interacts with the encoder through the attention mechanism to extract the contextual information of the input sequence from the encoder. The introduction of the self-attention mechanism is effective at capturing global dependencies, and it allows the model to take into account information from all the other positions in the sequence when calculating a certain position. For distant dependencies in chemical molecules or sentences, the self-attention mechanism is able to dynamically adjust the importance of each position through the distribution of weights, thus focusing on the relationships between the molecules in a reaction. For example, the reactive sites in some reaction representations may be far away from the groups they directly interact with, but through the self-attention mechanism, these long-distance dependencies can be accurately captured. Thus, the Transformer with an attention mechanism is a revolutionary model that can continue to lead the way in several machine learning areas.

### 2.3. Chemical Data Representation

**Figure 3:**
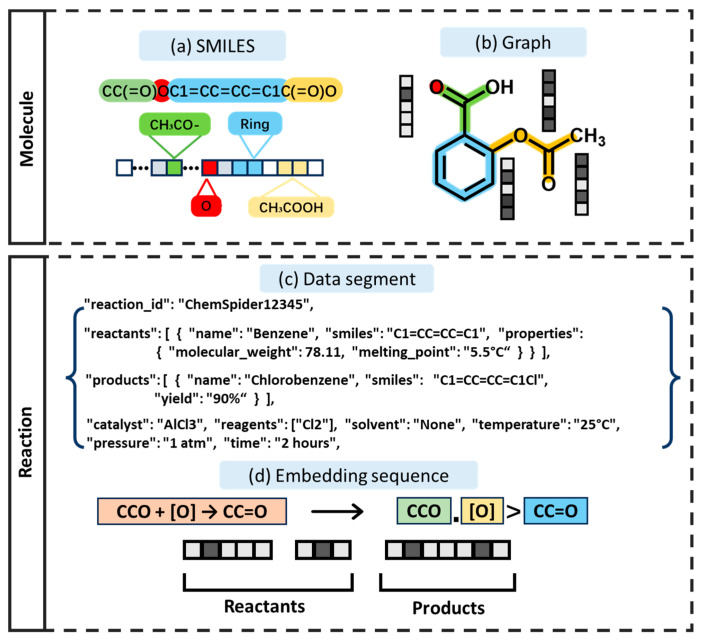
Molecular representation vs. reaction representation.

SMILES encodes the topology of a molecule using characters and symbols, as illustrated in Figure 3a. Compared with traditional molecular graph representations, SMILES is better suited for sequence modeling tasks in modern deep learning models, such as Transformers, and provides a straightforward and intuitive input format for chemical synthesis modeling [20]. By training on SMILES datasets, the Transformer effectively facilitates reaction prediction and retrosynthesis pathway planning [32]. Additionally, molecules can be represented as molecular graphs, with atoms as nodes and chemical bonds as edges, as shown in Figure 3b. The extended molecular graph structure can be adapted to the Transformer model by mapping features or designing specialized graph-based Transformers (e.g., Graph Transformers) to enhance the model’s ability to process graph data [28].

In chemical reaction databases, reactions are stored as key-value data segments that include various information about the reactions [33]. As shown in Figure 3c, chemical reactions are represented as SMILES string sequences. When input into a model, these sequences are embedded and encoded into numerical vectors based on their reaction relationships, enabling the model to recognize and process the reaction information [34]. This approach is applicable to both forward prediction (reactants → agents → products) and retrosynthesis (products → precursors), while also allowing for the integration of additional information, such as the reaction conditions and catalysts, to improve the prediction accuracy.

### 2.4. Reaction Datasets and Structuring

Chemical reaction datasets record information such as reactants, reagents, products, reaction conditions, reaction types, experimental yields, and infeasible reaction samples for model training. They often use SMILES or InChI [35] to represent the molecular structures and may apply data augmentation techniques to improve the model generalization. Mainstream datasets include USPTO, Reaxys, Open Reaction Database (ORD), and CAS SciFindern as shown in Table 1. USPTO is the most widely used reaction dataset in the public domain, and its data volume varies depending on the version and subset; the USPTO-50k dataset is a standard small-scale dataset containing 50,016 single-step reactions and is commonly used for model benchmarking; USPTO-full is a larger-scale dataset containing about 1,000,000 single-step chemical reactions; USPTO-MIT is processed to remove reagent and solvent information from the reactions and contains 479,035 single-step chemical reactions; and the USPTO-STEREO dataset contains about 1,800,000 reactions, which is the most widely used publicly available reaction dataset, depending on the version and subset. 800,000 reaction data with stereochemical information are preserved [36,37]. ORD is a publicly accessible resource that collects and standardizes detailed chemical reaction data to support research, reproducibility, and machine learning applications in reaction prediction and retrosynthesis [33]. Reaxys and CAS SciFindern are known for their high quality and comprehensive information on reaction conditions for industry and academic research [38]. In addition, some databases, such as Pistachio, focus on high-quality, open, and proprietary reaction data [39]. Researchers should select suitable datasets according to the task requirements, taking into account the data size, content quality, and whether it is publicly available, in order to support the efficient realization of chemical reaction prediction and pathway planning. High-quality structured datasets can be obtained through experimental data collection, text mining of the literature, patents, and databases [40]. Integrate cross and similar data from multiple sources by systematically organizing open-source reaction databases, and the negative reaction data (unfeasible reactions) can also be added in. This strategy reduces the data sparsity, enhances the coverage, and effectively differentiates feasible from unfeasible reactions, which will be able to improve the accuracy and reliability of chemical reaction predictions [41]. In addition, there are generally known and unknown reaction types in the dataset, which are determined by whether or not the dataset provides information on the type of reaction, e.g., nucleophilic substitution reactions and esterification reactions. Researchers used this to categorize the predictions of known and unknown reaction types to test the performance of the model more fully.

## 3. Transformer-Based Chemical Synthesis Applications

### 3.1. Retrosynthesis Pathway Planning

The main purpose of retrosynthesis prediction is to provide synthetic routes for target molecules to help chemists design experimental protocols and synthesize specific compounds, which is particularly important in drug discovery and development, making retrosynthesis planning a topic of extensive research. Retrosynthesis planning was first proposed by Corey et al. in 1969 [42]. In data-driven retrosynthesis prediction, one of the most significant approaches involves single-step prediction strategies for identifying precursor synthons of a target molecule. However, integrating pathway search algorithms with iterative precursor predictions enables the generation of longer retrosynthesis pathways until a commercially available starting material is identified [43,44,45]. Retrosynthesis methods are mainly categorized into template-based and template-free [46]. Synthetic pathways are constructed by breaking chemical bonds until the reaction is commercially available. Due to the wide range of possible reaction pathways, retrosynthesis planning can propose multiple synthetic routes and identify the optimal one through life cycle analysis or techno-economic evaluation, ultimately selecting a cost-effective pathway with minimal environmental impact.

The template method relies on predefined templates for known reactions in retrosynthesis planning, and it can provide predictions for known reaction types efficiently and quickly for situations where data are scarce or reaction rules are known. It is more interpretable but is unable to deal with novel or unknown reaction types and requires the construction of a large number of templates. The template-free method, on the other hand, does not rely on existing templates, and is able to generate innovative synthetic pathways by learning a large amount of reaction data, which is adaptable and especially suitable for unknown or complex reactions, but usually requires a large amount of training data and the interpretability of its results is poor as of now. Semi-template methods combine the advantages of template and template-free methods by combining partial template rules with a data-driven approach, which can provide efficient predictions while being innovative and adaptive, and are suitable for more diverse reaction types, but also face the challenge of balancing template selection and data training [47]. Currently based on the use of advanced deep learning, researchers are more focused on developing template-free retrosynthesis algorithms. As far as the application to the Transformer model is concerned, relatively more research was focused on the level of template-free methods. Most models based on single-step retrosynthesis deep learning use top-k accuracy measure. The top-k accuracy is the percentage of ground truth precursors in the top-k suggestions of a retrosynthesis model [48]. In retrosynthesis prediction, this is achieved by having the model generate k candidate reactions, ordered by the probability of the target product. The prediction is considered successful if the true combination of the reactants appears in the first n results predicted by the model. For example, when k is 1, the prediction is considered successful only if the model’s first candidate result is an exact match to the reactants recorded in the dataset.

In deep learning retrosynthesis prediction, single-step retrosynthesis prediction assumes that the target molecule is generated by a single-step chemical reaction, which does not involve complete reaction pathway planning, but only focuses on the direct relationship between the regenerated molecule and its reactants, and the output result is a set of candidate reactants. Karpov et al. [49], based on the Transformer model, transformed a compound’s SMILES string into another SMILES string representing its structural reactants. This transformation was used to predict the set of reactions required to synthesize the target molecule for retrosynthesis prediction. The model employs a recurrent learning-rate-scheduling strategy to train all parameters directly from the training set, combined with weight-averaging and snapshot-learning techniques. The researchers used a dataset that comprised 50,000 reactions filtered from the USPTO patent database, including 40,029 training samples, 5004 validation samples, and 5004 test samples. Ultimately, the model achieved a 42.7% top-1 accuracy on the test set, representing a 5.4 percentage point improvement over the original Seq2Seq method by Liu et al. [50], which also used a sequence model. This demonstrates that the Transformer model can be used effectively for retrosynthesis prediction tasks.

#### 3.1.1. Predictive Validity Improvement

However, models based on natural language processing face a common issue: the predicted reactant SMILES may be syntactically invalid, or the product and reactant may not form a chemically plausible reaction. To address the issues of an invalid output and unreliable predictions, Kim et al. [51] reported an approach that couples two Transformers: one for retrosynthesis reaction prediction and the other for forward reaction prediction. These models share most parameters and are better able to learn syntactic and retrosynthesis rules, thereby generating more accurate and syntactically valid predictions. The researchers introduced polynomial latent variables and learned their prior distributions to generate diverse candidate reactions. They also employed cyclic consistency checking to ensure the optimality of the predicted reactions. This method effectively tackles the problems of invalid SMILES and chemical infeasibility in template-free retrosynthesis methods. The method achieved a 47.1% top-1 accuracy and 78.5% top-10 accuracy on the USPTO-50K dataset with an unknown reaction type. However, the reactant candidates predicted by this method were repetitive and lacked diversity. Zheng et al. [26] proposed a template-free, self-correcting retrosynthesis prediction method. This method introduces a Transformer-based grammar corrector that automatically corrects invalid SMILES strings generated by the retrosynthesis predictor, leading to more reliable predictions. The grammar corrector constructs a training set of input–output pairs by using invalid ingredient candidates generated by the retrosynthesis predictor as inputs and the corresponding correct ingredients as outputs. The trained grammar corrector is coupled with the retrosynthesis predictor to effectively correct grammatical errors and significantly improve the prediction accuracy. On the USPTO-50K dataset, the model achieved a top-1 accuracy of 59.0%, which was over 6% higher than that of the template-based approach, and reduced the percentage of invalid predictions from 12.1% to 0.7%, which significantly increased the validity of the model output. On the more challenging USPTO-Full dataset, the model achieved an accuracy of 47.6% without including similar compounds from the training set. Schwaller et al. [32] combined the Molecular Transformer with an automated retrosynthesis-pathway-planning strategy using a hypergraph. The model performed well in predicting reactants, reagents, solvents, and catalysts in a single-step retrosynthesis task. The researchers modeled the retrosynthesis problem as a directed acyclic hypergraph based on a bayesian probabilistic approach, where each node represented a molecule, and each edge represented a reaction that connected multiple reactants and reagents. They used the single-step retrosynthesis Transformer to generate possible precursor molecules and combined the SCScore (Synthesis Feasibility Score) with the probability of predicting a positive reaction to rank and screen plausible reactions. They trained different retrosynthetic Transformer-based models with two different datasets: one fully based on open-source data (stereo) and one based on commercially available data from Pistachio (pistachio). And they were evaluated using a fixed forward prediction model (pistachio_i) on two validation sets (stereo and pistachio). More importantly, the researchers questioned the reliability of the top-k accuracy metrics. Traditional top-k accuracy metrics in retrosynthesis prediction primarily focus on identifying the expected precursor reactants, which fails to capture the model’s capability to predict chemically meaningful precursors. To address this limitation, researchers proposed four new metrics to evaluate the model performance: coverage, class diversity, round-trip accuracy, and Jensen–Shannon divergence, which are designed to emulate the expert evaluation system. Among these metrics, the coverage represents the percentage of desired products for which at least one valid precursor set was suggested. The class diversity reflects the diversity of the predicted reaction types. The round-trip accuracy assesses whether the predicted precursors can regenerate the original target molecule through a forward reaction. The inverse of the Jensen–Shannon divergence (1/JSD) quantifies the uniformity of the probability distribution across the predicted reaction types. The researchers trained different retrosynthetic Transformer-based models with two different datasets, one fully based on open-source data (stereo) and one on based commercially available data from Pistachio (pistachio), and evaluated both models using two validation datasets. The results show that both models had more than 90% coverage, with round-trip accuracies of up to 81.2%. The pistachio-based model demonstrated a higher 1/JSD score, suggesting that the probability distributions of the different reaction classes were more uniform, indicating reduced model bias. These evaluation metrics enable a more effective and comprehensive assessment of the value of retrosynthesis prediction for chemical applications.

#### 3.1.2. Predicting Generalizability Improvement

**Figure 4:**
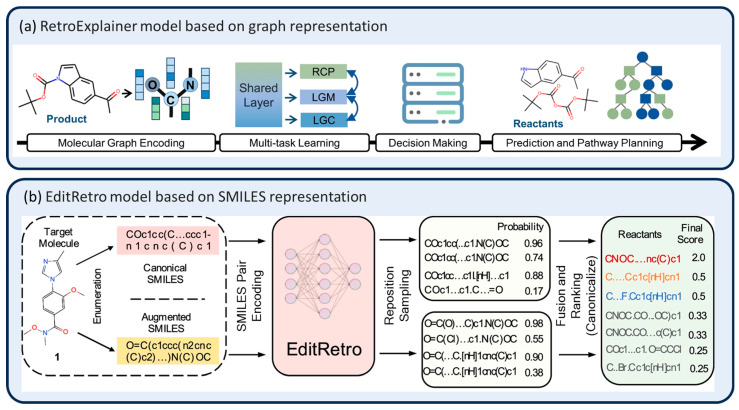
Schematic of RetroExplainer [55] and EditRetro [52] model retrosynthesis.

The performance of deep learning models depends on large amounts of high-quality data. Multi-task learning and data augmentation can help reduce model overfitting and improve the robustness, thereby enhancing the model’s generalization ability. Han et al. [52] proposed EditRetro, a retrosynthesis prediction model based on an enhanced molecular-string-editing task, as shown in Figure 4b. To achieve high-quality and diversified reactant predictions, EditRetro includes three key editing operations: sequence repositioning (adjusting the positions of atoms or groups in a molecule, e.g., reordering or deleting elements), placeholder insertion (predicting the positions where new atoms or groups should be inserted), and marker insertion (generating specific candidate reactant markers for the placeholders). The model consists of an encoder and three Transformer-based decoders. In each decoding iteration, the sequence relocation decoder identifies reaction centers and generates synthons, the placeholder decoder determines insertion positions, and the marker decoder generates specific reactant structures for the final output. Experiments showed that EditRetro achieved a top-1 exact match accuracy of 60.8% on the USPTO-50K dataset and 52.2% on the more challenging USPTO-FULL dataset, which demonstrated its efficiency and superior performance at handling complex chemical reaction tasks. By redefining the retrosynthesis task and introducing editing operations, EditRetro offers an innovative solution with significant advantages in generating efficient and diverse results. Chen et al. [53] introduced a discrete mixture model within the Transformer framework to encode different reaction types by adding latent variables. The model shares an encoder–decoder network, which sends latent variable embeddings as inputs to the decoder, ensuring that the outputs depend on the latent variables. During training, the Online Hard EM (Expectation Maximization) algorithm is used to select the latent variable value that minimizes the loss for each sample. This hard selection mechanism enables the model to learn diverse reaction types, thereby enhancing its generalization and diversity. On the standard USPTO-50K dataset, the hybrid model with five latent categories, combined with template pre-training and data enhancement, achieved a top-1 accuracy of 40.5% and a top-10 accuracy of 79.4%. However, on a test set that contained only “rare reactions,” the highest top-10 accuracy dropped to 39.9%, which suggests that the model still faces limitations in handling rare reactions. This resulted in a decrease of about 40 percentage points in accuracy compared with the full test set. In contrast, Tetko et al. [10] improved the Transformer performance through a data augmentation approach using randomized SMILES strings and molecular graphs. These SMILES strings represent valid compound structures, but the starting atoms of the molecular graph and the direction of graph traversal are randomly selected, thus enhancing both the input and target data. This method effectively mitigates the data-memory effect of neural networks and improves the prediction performance for new sequences. Despite the increased difficulty of using random SMILES as target data, the model was still able to memorize these sequences. On the USPTO-50K dataset, the top-1 accuracy of the method for unknown reaction types was 48.3%, suggesting that the data augmentation helped the model learn diverse SMILES representations and enhanced the generalization. However, the model may be less effective in practical applications where reactants and reagents cannot be separated, and its performance requires further optimization.

#### 3.1.3. Combined Molecular Graph

Since SMILES is a linear sequence representation and molecules are inherently graph structures (consisting of atomic nodes and chemical bonding edges), SMILES-based Transformer models may lose the topological information of molecules, especially when handling complex structures, such as cyclic or aromatic compounds. In contrast, graph-based Transformer models offer significant advantages in tasks, such as processing chemical molecules and retrosynthesis planning. Seo et al. [28] introduced a Transformer-based Graph Truncation Attention (GTA) model for retrosynthesis tasks. GTA utilizes the Transformer’s self-attention mechanism to incorporate molecular graph structural information into the self-attention layer, combining molecular sequences and graph representations to achieve dual modeling. In the encoder’s self-attention layer, GTA captures molecular graph structural information by using a graph-distance-based adjacency matrix as a mask, which allows for attention computation only between atoms whose graph distance is less than or equal to a set threshold. In the decoder’s cross-attention layer, the model uses atom-mapping information to guide the attention computation, focusing on relevant atoms in a chemical reaction. The model also introduces special handling for non-atomic markers in SMILES sequences, enabling them to perform attention calculations with all the markers to capture richer contextual information. GTA was in the top-1 and top-10 for accuracy at unknown reaction types at 51.1% and 81.6%, respectively, on the USPTO-50K dataset, and top-1 and top-10 accuracies of 46.0% and 70.0%, respectively, on the USPTO-Full dataset. Although GTA does not require completely accurate atom mapping, it still relies on some atom-mapping information, which must be obtained from chemical data using the FMCS algorithm, thereby increasing the workload. Mao et al. [54] also introduced molecular graph structures to be learned in a self-attention manner. The researchers proposed a graph-enhanced Transformer (GET) model that combines graph neural networks (GNNs) with both graphical and sequential representations of molecules. Their design of the GNN, called GAES, utilizes self-attention and bonding features to learn high-quality atomic representations. The inputs into GAES include 29-dimensional node features (chemical information) and 4-dimensional solo thermal-encoded edge features (chemical bond types). GAES learns node representations through an attention mechanism and a hopping structure, capturing higher-order neighbor information through stacking. In the model, the outputs of GAES and the Transformer encoders are directly spliced, fused using the attention mechanism, fused through the gating mechanism, and fused via linear transformation to generate the final encoder output. After the evaluation, GET-LT1 with linear transformation fusion performed the best, where it achieved a top-1 accuracy of 57.4% on the USPTO-50K dataset. When the reaction type information was removed, the top-1 accuracy of GET-LT1 still reached 44.9%. This suggests that the molecular structure information significantly improved the Transformer’s reactant prediction ability. However, the GET model only generates SMILES sequences for the output, which increases the likelihood of an invalid output. In addition, later, Wang et al. [55] proposed a Transformer-based model, RetroExplainer, as shown in Figure 4a, which is a variant model. It adopts a multi-perspective and multi-scale molecular-graph-encoding strategy using the multi-head attention mechanism to integrate important information from the chemical bonds and atomic topology. Additionally, it incorporates a multi-task learning (DAMT) strategy to simultaneously train three subtasks: reaction center prediction (RCP), leaving group matching (LGM), and leaving group connection (LGC). This approach effectively captures the reaction center, leaving group, and their connections. In the decision process, the model simulates retrosynthesis through five steps: identifying the reaction center and predicting changes in the chemical bonds and atoms; matching the leaving group and finding the corresponding leaving group in the database; connecting the leaving group to a fragment; selecting the optimal reaction path and optimizing the decision based on energy scoring; and generating the reactant to output the final product. This decision process provides a decision curve with energy scores, offering good interpretability. RetroExplainer achieved top-1 and top-3 accuracies of 66.8% and 88%, respectively, on the USPTO-50K dataset, and a top-1 accuracy of 51.4% on the USPTO-FULL dataset, where it outperformed other models and fully graph-based deep learning models at the time. The latest advancement in graph-binding-based Transformer models for retrosynthesis prediction is the Ualign model, proposed by Zeng et al. [25]. The researchers used a variant of the graph attention network to replace the encoder portion of the Transformer model, where they incorporated chemical bonding information into the message-passing process to create a more robust representation of the molecule. They also introduced an unsupervised SMILES alignment mechanism to establish associations between the product atoms and reaction SMILES markers, reducing the complexity of the SMILES generation and enabling the model to better learn chemical relationships. Finally, the Ualign model demonstrated excellent performance, where it achieved top-1 and top-3 accuracies of 66.4% and 86.7%, respectively, on the USPTO-50K dataset, and a top-1 accuracy of 53.5% on the test of unknown reaction types.

#### 3.1.4. Reaction Center Identification

**Figure 5:**
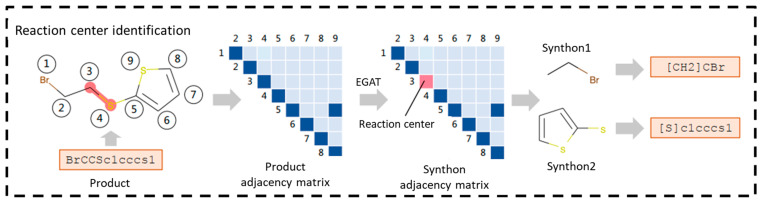
Graph-based identification of reaction centers [58].

The successful identification of reaction centers (i.e., regions of chemical bond breaking or reorganization) in retrosynthesis prediction will both make the predictions chemically plausible and significantly improve the prediction efficiency. Wan et al. [56] proposed the Retroformer model, which is based on the Transformer architecture and introduces a special local attention head that jointly encodes molecular sequences and molecular graphs. The global head is identical to the standard self-attention mechanism, covering the entire SMILES sequence, while the local head focuses on the molecular topology, with the scope of each token restricted to its one-hop neighborhood. The local head also incorporates bond vectors and bonding features into the computation through element-by-element multiplication. The model predicts the reaction probabilities $P_{rc}\left(s\right)$ and $P_{rc}\left(e\right)$ for each atom and bond through two fully connected layers: the atom reaction center identifier and the bond reaction center identifier. If a token exists in a reactive bond ($P_{rc}\left(e\right)$ > 0.5) and has its own reactivity ($P_{rc}\left(s\right)$ > 0.5), it is labeled as a reaction center. Subsequently, a subgraph search is performed on the molecular graph, and the highest-scoring subgraph is selected as the reaction center candidate. In the inference phase, the model generates k/n reactions for each candidate, which are ultimately sorted by the reaction center score and generation score. Retroformer achieved a top-1 accuracy of 64.0% and a top-10 accuracy of 88.3% on the USPTO-50K dataset. In the case of an unknown reaction type, the top-1 accuracy was 53.2%. Although Retroformer performs well in terms of molecular SMILES validity, the SMILES generation model may still modify molecular fragments unrelated to the reaction center, resulting in chemically meaningless outcomes. Additionally, Wang et al. [57] proposed a method named RetroPrime. This method first outputs the labels of the reaction center atoms of the target molecules directly from a Transformer model and generates “synthons” by predicting the locations of the chemical bond breaks. This label enhancement process helps the model identify potential reaction centers and simplifies the learning of complex reaction patterns. Subsequently, another Transformer model combines the predicted reaction centers from the previous stage for “tagging and alignment” to complete the reactant generation. This strategy enhances the model’s ability to model the conserved group relationships from “Products-to-Synthons” and “Synthons-to-Reactants” perspectives, thereby increasing the chemical confidence. RetroPrime achieved a top-1 accuracy of 64.8% on the USPTO-50K dataset and a top-1 accuracy of 51.4% when tested with an unknown reaction type. RetroPrime uses global SMILES as the input, and measures should be adopted to improve the chemical plausibility. Another molecular graph-based reaction center identification method is the algorithm RetroXpert, proposed by Yan et al. [58], which divides the retrosynthesis task into two steps. First, the potential reaction centers of the target molecules are identified by the Edge Enhanced Graph Attention Network (EGAT), as shown in Figure 5. Then, the reactants associated with the synthons are generated using the Transformer-based Reactant Generation Network (RGN). EGAT uses edge-embedded computational nodes and edge representations to predict the probability of chemical bond breakage and selects the most likely broken bonds to generate the reactants. Its edge-enhanced attention mechanism improves the accuracy of predicting the number of broken bonds, enhancing the overall retrosynthesis prediction performance, particularly in the absence of reaction-type information. Ultimately, EGAT achieved an 86.0% reaction center identification accuracy with reaction-type information and 64.9% without. For the retrosynthesis prediction, RetroXpert achieved top-1 accuracies of 62.1% with reaction class information, 51.4% without it, and 49.4% on the large-scale USPTO-Full dataset, demonstrating great performances. This also highlights the advantage of graph relations in reaction center identification.

#### 3.1.5. Combined Search Algorithms

Although single-step retrosynthesis prediction strategies were extensively studied and continuously improved, relatively few attempts were made to develop new algorithms for addressing the more complex and comprehensive retrosynthesis pathway prediction problems. Multi-step retrosynthesis pathway prediction can be achieved by iteratively applying the single-step model until a viable starting material is identified, thereby producing a complete retrosynthesis pathway. More importantly, the number of multi-step reactant combinations can be extremely large, leading to exponential growth in task complexity. To address this challenge in multi-node, long-range pathway prediction, complete retrosynthesis depends on advanced search algorithms to rationally plan synthetic routes [43,45]. Lin et al. [24] developed an automated retrosynthesis pathway planning system, AutoSynRoute, which integrates the Transformer architecture with Monte Carlo Tree Search (MCTS). This system effectively reproduces published synthetic pathways. Building on the success of single-step retrosynthesis prediction, the researchers implemented multi-step iterative retrosynthesis pathway planning and successfully synthesized four case products. Another Westerlund et al. [27] evaluated the performance of a transformation model called Chemformer for single-step and multi-step retrosynthesis prediction. Chemformer is a Transformer-based retrosynthesis model that employs the BART architecture to process molecular SMILES strings, targeting both single-step and multi-step retrosynthesis tasks. For multi-step retrosynthesis, Chemformer integrates MCTS to optimize pathway planning by recursively invoking the single-step retrosynthesis model to iteratively generate precursors and starting materials for the target molecule. MCTS dynamically generates nodes, predicts candidate molecules in batches, and caches results to reduce redundant computations, significantly enhancing the pathway generation efficiency. The model was trained on a proprietary dataset that comprised approximately 18 million literature, patent, and experimental records. The researchers tested the template-based Chemformer in comparison with the template-free Chemformer, and the template-free one was able to obtain at least one route to retrosynthesis for 95% of the target molecules, whereas the template-based one resolved the synthesis routes for only 72% of the target molecules, and these predicted multi-step retrosynthesis routes corresponded to commercially available starting materials.

The application of the Transformer model in retrosynthesis benefits from its sequence-modeling capabilities, making it particularly well suited for template-free retrosynthesis prediction. Compared with other methods, those based on various molecular attention representations show superior prediction performance. Additionally, techniques such as correction, reaction center identification, and data enhancement can further improve the model’s predictive ability, as shown in Table 2, with the RetroExplainer and Ualign models exhibiting particularly significant performance.

### 3.2. Prediction of Forward Chemical Reactions

In chemical synthesis research, reaction prediction involves forecasting potential products based on known reactants and their associated conditions. Unlike retrosynthesis, the primary goal is to generate accurate product predictions by modeling the interactions between reactants and the influence of reaction conditions on the reaction pathway, which is crucial for advancing fundamental chemical research and understanding reaction mechanisms. Within the Transformer framework, reaction prediction is also treated as a sequence-to-sequence transformation problem. However, unlike the retrosynthesis task, which involves a one-to-many mapping relationship, reaction prediction is a many-to-one problem. This makes it particularly well suited for tasks involving SMILES string processing.

**Figure 6:**
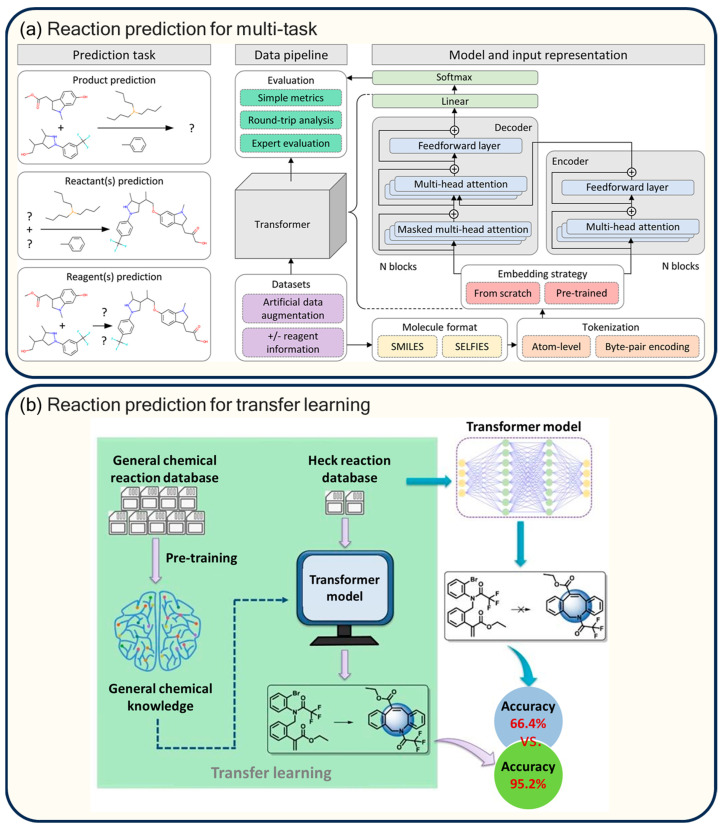
(**a**) Multi-task reaction prediction [60] and (**b**) transfer learning improved [21] by Transformer.

The Molecular Transformer model proposed by Schwaller et al. [17] uses SMILES strings to represent reactants, reagents, and products, and utilizes a multi-head self-attention mechanism to capture global dependencies in reaction SMILES sequences, the model is particularly adept at modeling the interactions between remote atoms or groups. Experimental results show that Molecular Transformer achieved a 90.4% top-1 accuracy on the USPTO-MIT dataset, and excelled in stereochemistry, selectivity, and other areas. Later, Schwaller et al. [59] enhanced the Molecular Transformer to develop the BERT (Bidirectional Encoder Representations from Transformers) model for reaction classification prediction. This improvement involves unsupervised pre-training using the masked language model loss function. The introduction of multifunctional representations of reaction fingerprints further allowed the model to explore and visualize the chemical reaction space in novel ways. Ultimately, the BERT model achieved a reaction classification accuracy of 98.2% on the Pistachio dataset, albeit relative to manually labeled reaction classes, and 98.9% on the USPTO 1k TPL test set. Qiao et al. [60] proposed a multi-task learning-based Transformer architecture designed to address the challenges of low-resource chemical reaction prediction. The researchers developed two multi-task models: the MFRPT (Multi Forward Reaction Prediction Transformer) and the RFRPT (Retrosynthesis and Forward Prediction Combined Transformer). The MFRPT model extracts shared features across various reaction datasets by using a single encoder while providing each task (e.g., Baeyer–Villiger oxidation, Heck reaction, Chan–Lam coupling) with an independent decoder and embedding layer. The RFRPT, on the other hand, treats forward prediction and retrosynthesis prediction as distinct tasks, employing a shared encoder and independent decoders to facilitate the comprehensive learning of chemical reaction principles by leveraging shared representations of source and target molecules. Using three low-resource reaction datasets (Baeyer–Villiger oxidation, Heck reaction, and Chan–Lam coupling) from the Reaxys database, experimental results reveal that the top-1 accuracies of the MFRPT and RFRPT were 75.7% and 72.1% on the Baeyer–Villiger dataset, 81.0% and 80.4% on the Heck dataset, and 83.0% and 78.2% on the Chan–Lam dataset. These results highlight the advantages of Transformer-based architectures in multi-task learning for chemical reactions. Additionally, Wang et al. [21] employed transfer learning for Heck reaction prediction using a small dataset of chemical data, as illustrated in Figure 6b. The researchers first pre-trained a Transformer model on a large database of chemical reactions to acquire general knowledge of the chemical reactions. They then fine-tuned the pre-trained model on a small Heck reaction dataset to improve the prediction accuracy for the Heck reaction. Five sets of training, validation, and test datasets were randomly generated to evaluate the performance of both the baseline Transformer model and the transfer learning model. In the overall Heck reaction prediction task, the transfer learning model achieved a top-1 accuracy of 94.9%, where it significantly outperformed the baseline model’s 66.3%. For subtypes of the Heck reaction (i.e., intermolecular and intramolecular Heck reactions), the transfer learning model achieved top-1 accuracies of 95.3% and 87.7%, respectively, compared with 66.7% and 58.7% for the baseline model. Furthermore, the researchers analyzed the model’s performance in predicting Heck reactions involving different substituted olefins, and the results were consistent with the kinetic laws of the chemical reactions.

The performance of reaction prediction can be significantly improved by utilizing data augmentation or custom editing tasks for SMILES. Jaume-Santero et al. [61] proposed a methodology to analyze the performance of the Transformer model in chemical reaction prediction tasks, including product, reactant, and reagent prediction, as shown in Figure 6a, where the researchers used a non-standard SMILES representation and enhanced the data by randomizing reactants and reagents to generate alternative versions of the reaction. Additionally, two input formats, SMILES and SELFIES, were used, and comparative analyses of labeling strategies were conducted at the atomic level using byte pair encoding. Using the USPTO-MIT dataset, the model achieved an 88% top-1 accuracy for reaction prediction with reagent information and 87% accuracy, even without reagent information. Zhong et al. [62] proposed an R-SMILES model, which innovatively uses the same root atoms to represent the SMILES strings of both the reactants and products, ensuring a tight one-to-one mapping between the inputs and outputs. This approach eliminates the potential diversity in generic SMILES representations and allows the model to focus on learning chemical reactions rather than complex SMILES syntax. On the USPTO-50K, USPTO-MIT, and USPTO-FULL datasets, R-SMILES reduced the minimum edit distance between the input and output SMILES by 21%, 21%, and 16%, respectively. The advantage of R-SMILES was particularly significant for neo-atomic or chiral complex reactions, with a 39.3% improvement in accuracy for neo-atomic reactions and only a 4.3% decrease in accuracy for chiral reactions when R-SMILES was used, compared with a 13.3% decrease without it. While incorporating molecular graph information generally eliminates the need for SMILES data augmentation and simplifies the data processing, Tu et al. [63] proposed a novel architecture called Graph2SMILES. This combines the benefits of the Transformer model for text generation with the alignment invariance of the molecular graph encoder. The researchers employed a directed message-passing neural network (D-MPNN) to capture the local chemical context of the molecule, with a focus on directed bonds to avoid the cyclic transfer of messages between neighboring atoms. This was combined with an attention mechanism to improve the capture of the local chemical context by aggregating messages from neighboring atoms, which laid the foundation for global encoding and molecular transformation tasks, and the architecture was applied to retrosynthesis tasks. In reaction prediction, the Graph2SMILES model achieved a 90.3% top-1 accuracy on the USPTO_480k_mixed dataset and a 78.1% top-1 accuracy on the USPTO_STEREO_mixed dataset. For the single-step retrosynthesis task, Graph2SMILES achieved top-1 accuracies of 45.7% and 52.9% on the USPTO_full and USPTO_50k datasets. The Transformer model is flexible enough to accommodate both forward and reverse directions for predictable reactions and retrosynthesis multi-tasking predictions. Lu et al. [64] proposed a model called T5Chem, developed based on the Text-To-Text Transfer Transformer (T5) architecture. Two major modifications were made to the T5 compared with the original Transformer model: (1) removal of the layer normalization bias and placing normalization outside the residual paths, and (2) the use of relative positional encoding instead of sinusoidal positional encoding. T5Chem was pre-trained on 97 million PubChem molecules using masked language modeling (MLM), which resulted in better initialization and faster convergence. T5Chem was pre-trained on 97 million PubChem molecules using the Masked Language Model (MLM), which resulted in improved initialization and faster convergence. The model is suitable for a variety of chemical reaction prediction tasks, including reaction type classification, forward reaction prediction, single-step retrosynthesis, reaction yield prediction, and reagent prediction. By training on the multi-task dataset USPTO_500_MT, T5Chem facilitates mutual learning between tasks, thereby improving its robustness. Ultimately, the model achieved a 97.5% top-1 accuracy in the forward reaction prediction task, 99.4% in the reaction-type classification task, and 72.9% top-1 accuracy in the single-step retrosynthesis task. The forward reaction prediction was significantly less complex than the retrosynthesis, and using the forward reaction predictions to correct the retrosynthesis predictions improved both the reasonableness of the retrosynthesis predictions and the generalization of the model.

## 4. Discussion and Outlook

In discussing the application of the Transformer in chemical synthesis, we summarized its numerous applications in chemical reactions and retrosynthesis, highlighting the excellent performance of the Transformer framework. These include simplifying sequential tasks, encoding specific domains, scalability, and multimodality, all of which can further enhance the performance and address more complex tasks.

### 4.1. Extended Model

The Transformer model, as a data-driven generative model, captures global dependencies in molecules but lacks an intrinsic understanding of specific chemical rules. This limitation can result in the generation of ineffective reactions or irrational molecular structures [26]. While it is possible to train models on data to discover patterns of reactions, these patterns may not necessarily be consistent with chemical theory. For example, important chemical concepts, such as reaction centers, transition states, and molecular orbitals, are not natural mechanisms for Transformer models to understand and apply. Therefore, how to combine the expertise in the field of chemistry (e.g., reaction rules and reaction mechanisms) with the Transformer architecture is an urgent problem to be solved. Meanwhile, a Transformer has some limitations in local structure modeling. The key to many chemical reactions lies in the local structure changes (e.g., reaction center identification), and a Transformer’s ability to model local information is relatively weak. Especially in tasks such as reaction center identification and leaving group localization, how to make a Transformer better focus on key regions in molecules remains an important research question. And in the task of chemical reaction prediction, especially for some rare reactions or new types of chemical reactions, there is a lack of sufficient training data. This makes a Transformer’s performance less stable in the face of small samples or uncommon reactions. The problem of data scarcity may lead to model overfitting or poor generalization.

**Figure 7:**
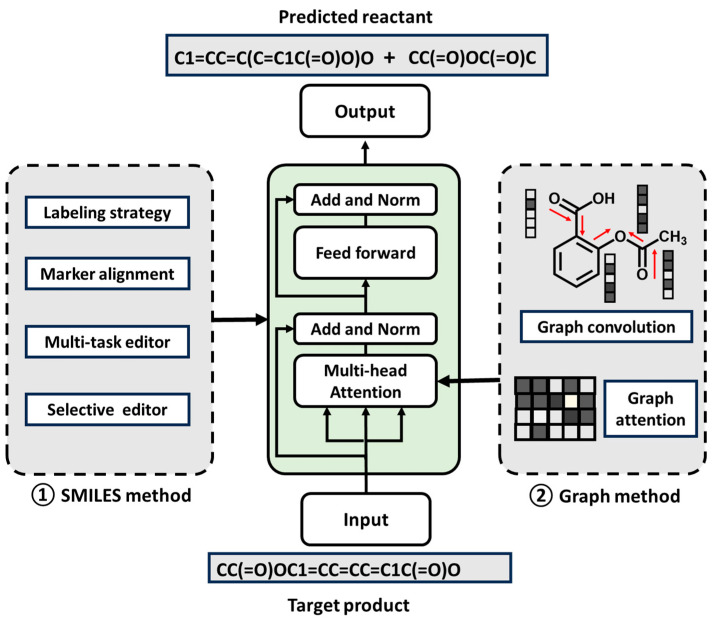
Strategy for the SMILES task combined with the molecular graph strategy.

According to existing research, on one hand, pre-existing chemical knowledge tags can be embedded into the Transformer’s generation process using the rule-embedding approach, which ensures that the generated results conform to known chemical rules. This approach is particularly effective in retrosynthesis planning, where it helps filter candidate reactions to ensure chemical rationality. On the other hand, it can be combined with a graph neural network (GNN) to optimize the molecular relationship modeling. Numerous studies demonstrated the effectiveness of this approach, and by combining a GNN with a Transformer, the model can more efficiently learn the structural information of molecules. Additionally, using molecular graph embedding or graph-based reaction center recognition to enhance local attention can significantly improve the model’s predictive performance [63]. Reaction-rule-based filters were also used in conjunction with molecular features extracted by GNNs to screen for chemically valid candidate molecules, which enhanced the model’s effectiveness in both local and global learning. As shown in Figure 7, the fully SMILES-based strategy incorporates expert knowledge during the editing task to improve predictions, while the strategy that combines molecular graphs utilizes graph convolutional information or graph attention mechanisms to enhance the learning of molecular rules. The comparison in this paper reveals that combining these two approaches generally leads to superior predictions. Also using forward reaction prediction and reverse retrosynthesis to correct each other is more likely to improve the model performance. For “rare reactions” or small sample datasets, data augmentation or transfer learning can effectively address this problem. Therefore, based on the scalability and multimodality of a Transformer, it can be predicted that hybrid modeling with mixed-input multi-tasking and other prediction strategies can significantly enhance model performance, enabling the model to generalize more effectively to different reaction types.

**Table 2:** The top-k accuracy of retrosynthesis on USPTO-50K for the main method applied by Transformer.

Model	Year	Top-k Accuracy (%)					
		Reaction Class Known			Reaction Class Unknown		
		1	3	5	1	3	5
Template-based							
NeuralSym [9]	2017	55.3	76.0	81.4	44.4	65.3	72.4
Semi-template-based							
RetroXpert [58]	2020	62.1	75.8	78.5	50.4	61.1	62.3
RetroPrime [57]	2021	64.8	81.6	85.0	51.4	70.8	74.0
Template-free							
SCROP [26]	2020	59.0	74.8	78.1	43.7	60.0	65.2
Aug. Transformer [10]	2020	-	-	-	48.3	-	73.4
Tied Transformer [51]	2021	-	-	-	47.1	67.1	73.1
GTA [28]	2021	-	-	-	51.1	67.6	74.8
Graph2SMILES [64]	2022	-	-	-	52.9	66.5	70.0
GET [54]	2021	57.4	71.3	74.8	44.9	58.8	62.4
Retroformer [56]	2022	64.0	82.5	86.7	53.2	71.1	76.6
RetroExplainer [55]	2023	66.8	88.0	92.5	57.7	79.2	84.8
Ualign [25]	2024	66.4	86.7	91.5	53.5	77.3	84.6

### 4.2. Limitations in Dataset

The foundation of the chemical reaction and retrosynthesis prediction lies in a model’s understanding of chemical reaction mechanisms, with high-quality datasets being essential for advancing research in this field. However, existing benchmark datasets (e.g., USPTO-MIT and USPTO-50K) suffer from significant limitations, which create multiple challenges for chemical reaction prediction and retrosynthesis modeling. The distribution of reaction types in the USPTO-MIT dataset is highly unbalanced, with linear topology reactions dominating the dataset, while reactions with cyclic topologies are significantly underrepresented [65]. This leads to a tendency for models to perform well on high-frequency reaction types while underpredicting low-frequency reactions, such as those involving rare cyclic topologies. USPTO-50K, the primary benchmark dataset for retrosynthesis prediction, contains only 50,000 reverse reaction records. Given the minimal differences in top-k accuracy between the current methods, this small-scale dataset is no longer adequate to fully evaluate the true capability of these models. Moreover, the limited size of the dataset may restrict the model’s generalization ability, making it challenging to maintain robustness in complex chemical spaces. Additionally, the USPTO benchmark dataset excludes information on by-products in reaction prediction and retrosynthesis tasks, focusing solely on major products. This simplification may compromise the modeling of complete chemical reactions, particularly in scenarios involving complex chemical equilibria or side reactions. While the omission of by-products may not significantly affect single-step retrosynthesis predictions, their role in multi-step reaction chains remains critical and cannot be overlooked. Current solutions to these challenges often include data augmentation techniques (e.g., generating synthetic data for rare reaction types), modifying sampling strategies during training to address data imbalance, or using transfer learning methods to apply knowledge from high-frequency reactions to rare reactions. These approaches are often combined with small-sample learning methods to enhance the model’s adaptability to low-frequency reactions.

### 4.3. Molecular Conformational Information

In the application of chemical synthesis, Transformer models based on sequence modeling typically give minimal consideration to the conformational information of molecules. Even when molecular diagram information is incorporated, it is often limited to a 2D representation [28]. A molecule can adopt multiple conformations, and certain reaction sites may be obscured due to the steric hindrance caused by its 3D conformation. Additionally, the 3D structure may influence the electron density distribution, altering the reactivity and properties of these sites. In current Transformer-based molecular representation and generation models, related studies explored conformational generation. Some approaches integrate Transformer models with 3D spatial information, selecting key spatial atoms using sampling algorithms assisted by attention mechanisms. Others utilize a combination of atomic position encoding and distance encoding in both 2D and 3D spaces. These models effectively capture and characterize the spatial properties of molecules [66,67]. Some conformational sampling tools (e.g., RDKit) were utilized to quickly sample molecular conformations, select a range of possible conformations, or generate statistical distributions to serve as input features for models. Furthermore, molecular conformation is influenced by its environment (e.g., solvent). To account for this, it is advisable to incorporate atomistic simulation methods with low computational costs, such as molecular dynamics simulations, to generate conformational information of molecules in various environments. Representative conformations can then be selected as model inputs, or conformationally averaged features can be used to represent molecular information in three dimensions. Additional methodological advancements can be achieved by leveraging innovative approaches in molecular generation models for incorporating conformational information, in conjunction with the predictive modeling processes for chemical synthesis.

### 4.4. Multi-Task Learning

Multi-task learning has great potential for chemical reaction prediction and retrosynthesis prediction. By jointly optimizing multiple related tasks on shared spatial information, collaborative learning and knowledge transfer between tasks can be achieved, which significantly improves the prediction performance and generalization ability of the model. Within this framework, tasks such as reaction classification, reaction yield prediction, reaction site prediction, and reaction condition optimization can be jointly modeled, along with reaction prediction and retrosynthesis prediction. This approach provides a comprehensive understanding of chemical reactions from multiple perspectives. For example, reaction classification tasks can provide a priori knowledge about reaction types for prediction, and quantitative predictions, such as reaction yields and selectivity, are essential for determining the cost-effectiveness of synthetic routes, which has an important role in multi-step synthesis. The Transformer model utilizes a self-attentive mechanism and flexible multimodal input processing capability that is scalable and allows for the efficient sharing of characterization information, such as molecular structure, reaction conditions, and product distribution, to capture potential chemical correlations across tasks [61]. While multi-task learning frameworks face challenges such as task optimization conflicts and data imbalances, these issues can be mitigated through techniques like dynamic weight assignment strategies and self-supervised pre-training. In the future, multi-task learning is expected to further enhance the intelligence and utility of chemical reaction and retrosynthesis predictions, offering efficient and accurate tools for the design of complex molecules.

## 5. Conclusions

This paper reviews the applications and progress of the Transformer model and its improved variants in the field of chemical synthesis. The Transformer model has greatly improved the efficiency and accuracy of chemical reaction prediction and retrosynthesis pathway planning by virtue of its powerful sequence modeling capability, self-attention mechanism, and strong extensibility. Its various variants were widely used in single- and multi-step retrosynthesis planning, forward reaction prediction, etc., and showed great potential in complex molecule synthesis and high-throughput screening. Despite these achievements, there are still some challenges that need to be addressed, including the model’s deep understanding of chemical rules, the interpretability of the generated results, and dataset limitations. Future research may focus on combining a Transformer with more chemically empirical techniques to further explore its application potential. In addition, optimizing cross-modal data integration and enhancing multi-task learning frameworks are also key directions to advance the application of Transformer models in chemical synthesis. In addition, the integration of reinforcement learning and knowledge graphs can enable the Transformer to play a more prominent role in the fields of green chemical design, complex molecule synthesis, and drug discovery, injecting a new impetus to the intersection of modern chemistry and artificial intelligence.

## Figures and Tables

**Table 1:** Summary of main chemical reaction datasets.

Database	Data Volume	Data Sources	Open Source	Address *
Reaxys	64 million	Integration of Beilstein and Gmelin databases, patent chemistry databases from journals, patents, etc.	No	https://www.reaxys.com
CAS SciFindern	150 million	Sourced from more than 10,000 journals and 64 patent offices around the world, including Patent Markush Structures.	No	https://scifinder-n.cas.org
USPTO Reaction Database	50,000 (USPTO-50k) 1 million (USPTO-full) 480,000 (USPTO-MIT) 1.8 million (USPTO-STEREO)	Data from U.S. Patent Literature, which is free and open.	Yes	https://tdcommons.ai/generation_tasks/retrosyn/#uspto-50k https://github.com/wengong-jin/nips17-rexgen/tree/master/USPTO
Open Reaction Database	2 million	Sourced from journals, patents, and experimental data, supporting researcher uploading and sharing.	Yes	https://docs.open-reaction-database.org/en/latest

* The above database URL access date is 31 December 2024.

## Data Availability

Not applicable.
